# An integrative multi-omics analysis based on disulfidptosis-related prognostic signature and distinct subtypes of clear cell renal cell carcinoma

**DOI:** 10.3389/fonc.2023.1207068

**Published:** 2023-06-23

**Authors:** Dong Zhang, Xi Zhang, Zhanpeng Liu, Tian Han, Kai Zhao, Xinchi Xu, Xu Zhang, Xiaohan Ren, Chao Qin

**Affiliations:** The State Key Lab of Reproductive, Department of Urology, The First Affiliated Hospital of Nanjing Medical University, Nanjing, China

**Keywords:** disulfidptosis, clear cell renal cell carcinoma, prognosis, immunity, SLC7A11

## Abstract

**Background:**

The association between clear cell renal cell carcinoma (ccRCC) and disulfidoptosis remains to be thoroughly investigated.

**Methods:**

We conducted multiple bioinformatics analyses, including prognostic analysis and cluster analysis, using R software. Additionally, we utilized Quantitative Real-time PCR to measure RNA levels of specific genes. The proliferation of ccRCC was assessed through CCK8 and colony formation assays, while the invasion and migration of ccRCC cells were evaluated using the transwell assay.

**Results:**

In this study, utilizing data from multiple ccRCC cohorts, we identified molecules that contribute to disulfidoptosis. We conducted a comprehensive investigation into the prognostic and immunological roles of these molecules. Among the disulfidoptosis-related metabolism genes (DMGs), LRPPRC, OXSM, GYS1, and SLC7A11 exhibited significant correlations with ccRCC patient prognosis. Based on our signature, patients in different groups displayed varying levels of immune infiltration and different mutation profiles. Furthermore, we classified patients into two clusters and identified multiple functional pathways that play important roles in the occurrence and development of ccRCC. Given its critical role in disulfidoptosis, we conducted further analysis on SLC7A11. Our results demonstrated that ccRCC cells with high expression of SLC7A11 exhibited a malignant phenotype.

**Conclusions:**

These findings enhanced our understanding of the underlying function of DMGs in ccRCC.

## Introduction

Nowadays, renal cell carcinoma (RCC) has become one of the most prevalent genitourinary tumors with a high mortality rate (1). RCC comprises three major histological subtypes, among which ccRCC accounts for 80 to 90% (2). Despite contemporary improvements in multiple new therapies, the prognosis of patients remains poor since early-stage ccRCC is usually asymptomatic (3). Previous research has proposed several scoring systems based on stage, size, and grade for prognostic estimation of ccRCC patients (4). However, these scoring systems heavily rely on clinical parameters and do not consider molecular markers, which have implications in choosing appropriate therapeutic strategies. Therefore, there is an urgent need to identify reliable and novel prognostic molecular biomarkers to advance precision prognosis and improve outcomes for ccRCC patients.

The process of gene-regulated cell death, known as “programmed cell death,” is of great significance for tissue growth, homeostasis, and several pathological activities (5). Studies have demonstrated that cell death plays a role in tumorigenesis, tumor progression, metastasis, drug resistance, and prognosis (6). More recently, a novel cell death modality called disulfidoptosis has been discovered, distinct from other programmed cell death processes (7). Disulfidoptosis occurs due to disulfide stress, which is caused by the abnormal accumulation of disulfides and can be highly toxic to cells, affecting cancer cell survival and proliferation (8). Disulfide metabolism also influences biological activities associated with cancer cells. A previous study revealed the association between disulfide nanoparticles contained in convection-enhanced delivery and resistance to the chemotherapeutic temozolomide in patients with glioblastoma (9). Disulfidoptosis may become a research hotspot in the future for cancer immunotherapy, and recent research has shown the potential of targeting disulfidoptosis for treating bladder cancer (10). Several genes involved in disulfidoptosis have been identified, providing researchers with opportunities to predict the prognosis of ccRCC patients. However, the prognostic and therapeutic role of these genes in ccRCC has not yet been investigated.

The rapid development of bioinformatics has provided great convenience for researchers (11–13). In this study, we identified that the expression of specific DMGs was associated with different prognosis outcomes. We then comprehensively investigated the clinical relevance and underlying biological role of these genes. Furthermore, considering the importance of SLC7A11 in disulfidoptosis, we performed experiments to verify the function of SLC7A11 in ccRCC. Our findings demonstrated the oncogenic function of SLC7A11 in promoting the migration, invasion, and progression of ccRCC cells.

## Methods

### Data acquisition and processing

The consolidated transcriptome expression matrix and clinical data of ccRCC were obtained from the Cancer Genome Atlas (TCGA) and Gene Expression Omnibus (GEO) databases. To validate our findings, we utilized the GSE22541 dataset as the external validation dataset. Additionally, the GSE17895 and GSE73731 datasets were used to validate the clinicopathological characteristics of SLC7A11 in ccRCC samples. In a related review, a total of 10 DMGs including SLC7A11, SLC3A2, NUBPL, NDUFA11, LRPPRC, OXSM, NDUFS1, GYS1, RPN1 and NCKAP1 were summarized (7).

### Construction of disulfidptosis metabolism-related signature

We performed Cox regression analysis with a significance threshold of P-value < 0.05, to identify candidate prognosis-related DMGs. Subsequently, we employed the least absolute shrinkage and selection operator (LASSO) Cox regression and multivariate Cox regression analysis to construct the disulfidoptosis metabolism-related signature (DMS). The algorithm used for constructing the DMS was as follows: DMS = Coef A * Gene A expression + Coef B * Gene B expression +… + Coef X * Gene X expression, where Coef represented the coefficient calculated by multivariate Cox regression and gene expression referred to the expression levels of the DMGs. The patients were then divided into either the low DMS group or the high DMS group based on the median DMS value. The prognostic accuracy of the DMS was evaluated using Kaplan-Meier (KM) analysis, the area under the curve (AUC) of the receiver operating characteristic (ROC) curve, as well as univariate and multivariable logistic regression analyses.

### Analysis of immune infiltration and function

Single-sample GSEA was performed using the “GSVA” package to calculate enrichment scores for different immune cell types and immunologic functions using immune-related gene sets. The immune activity scores of ccRCC samples were obtained from the Tracking Tumor Immunophenotype (TIP) database (http://biocc.hrbmu.edu.cn/TIP/). Immunotherapy data for ccRCC were downloaded from the Cancer Immunome Atlas (TCIA). Subsequently, differences in immunotherapy between the groups were further analyzed.

### Mutation and drug sensitivity analysis

Differences in somatic mutations were analyzed using the “maftools” R package, and the expression of tumor mutational burden (TMB) was compared between the two groups. The KM curve was used to assess the difference in survival between the mutation and the DMS combination. In the analysis of targeted therapy drugs, the “pRRophetic” package was employed to evaluate the IC50 values of nine commonly used chemotherapy drugs for renal cancer (Bosutinib, Gefitinib, Nilotinib, Pazopanib, Rapamycin, Sunitinib, Vorinostat, Tipifarnib, and Temsirolimus).

### Construction of the disulfidptosis metabolism-related clusters and bioinformatics analysis

We utilized the ConsensusClusterPlus package to identify different disulfidptosis modification patterns and classify patients for further investigation. KM analysis was performed to explore the survival differences between the disulfidptosis metabolism-related clusters. We then screened for differently expressed genes (DEGs) between the clusters based on criteria of |logFC| >= 2 and adj P Value < 0.01. Based on the expression of these DEGs, we evaluated the enrichment of functional biological pathways using Gene Ontology (GO) and Kyoto Encyclopedia of Genes and Genomes (KEGG). Furthermore, Gene Set Variation Analysis (GSVA) was conducted to assess the enrichment of cancer-related pathways between the different disulfidptosis metabolism-related clusters using the “h.all.v2023.1.Hs.symbols” gene set from the MSigDB.

### Quantitative real-time PCR

Human ccRCC cell lines (ACHN, OSRC-2, Caki-1, and 786-O) and normal renal epithelial cell line (HK-2), purchased from the laboratory, were used for quantitative reverse transcription polymerase chain reaction (qRT-PCR). Total RNA was extracted using TRIzol reagent (Thermo Fisher Scientific, Waltham, MA, United States). Reverse transcription of RNA into cDNA was performed using a reverse transcription kit (Vazyme #R333, Nanjing, China). The qPCR assay was conducted using SYBR-Green methods. The primer sequences used for qRT-PCR were as follows:

SLC7A11:
 - Forward primer: 5’-GGTCCATTACCAGCTTTTGTACG-3’
 - Reverse primer: 5’- AATGTAGCGTCCAAATGCCAG -3’
GAPDH:
 - Forward primer: 5’-CACCAGGGCTGCTTTTAACTCTG-3’
 - Reverse primer: 5’-GATTTTGGAGGGATCTCGCTCCTG-3’.

### Cell proliferation assay and Transwell assay

Following the standard procedure, the proliferation ability of the cells was assessed with CCK8 and colony formation assays. As per standard procedures, cells expressing si-SLC7A11, and control cells were transwell assayed.

### Statistical analysis

All analyses were conducted using R 4.2.2. We used a two-sided test for analysis, and P-value <0.05 was defined as statistical significance, unless otherwise noted. The KM curve and log-rank tests were utilized to evaluate the correlation between DMGs and overall survival (OS) in ccRCC patients.

## Results

### Construction of the disulfidptosis metabolism-related signature

**Figure 1:**
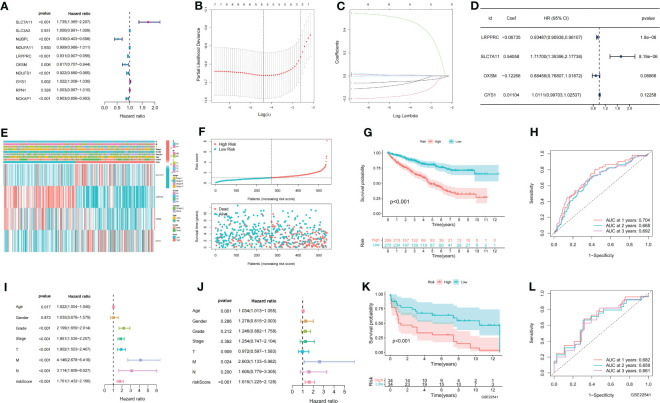
Establishment of disulfidptosis related signature. **(A)** Univariate COX results for 10 Disulfidptosis related genes; **(B)** LASSO coefficient profiles of the expression of 7 prognostic Disulfidptosis related genes; **(C)** Selection of the penalty parameter (λ) in the LASSO model *via* 10-fold cross-validation; **(D)** Multivariate COX results for 4 Disulfidptosis related genes; **(E)** The distribution of modeled gene expression and clinicopathological variables in the Disulfidptosis related signature; **(F)** The risk curve of each sample reordered by Disulfidptosis related signature and the scatter plot of the sample survival overview. The green and red dots represent survival and death, respectively; **(G)** KM curve showing the prognostic difference between high and low risk groups; **(H)** ROC curves about Disulfidptosis related signature in 1,2,3 years; **(I, J)** The univariate and multivariate Cox regression analysis of riskscore, age, gender, grade, stage, TMN stage; **(K)** The KM survival curve of Disulfidptosis related signature in the GSE22541 data set; **(L)** ROC curves about Disulfidptosis related signature in 1,2,3 years in the GSE22541 cohort.

A total of seven prognostic DMGs (SLC7A11, NUBPL, LRPPRC, OXSM, NDUFS1, GYS1, and NCKAP1) were identified through univariate Cox analysis (**Figure 1A**). Five DMGs were further analyzed using Lasso regression (**Figures 1B, C**). Finally, four DMGs were selected to establish the DMS through multivariate Cox regression analysis (**Figure 1D**). The expression profiles and clinicopathological features of the four modeled genes were presented in a heat map (**Figure 1E**). The distribution of DMS, survival status, and the KM survival curve demonstrated a positive association between DMS and mortality (**Figures 1F, G**). The ROC curves for DMS at 1, 2, and 3 years in the TCGA dataset were 0.704, 0.668, and 0.692, respectively (**Figure 1H**). Cox analysis further confirmed the high accuracy of DMS in predicting the prognosis of ccRCC patients (**Figures 1I, J**).

To validate the accuracy of DMS, we utilized the GSE22541 dataset as an external validation set. The KM curve demonstrated that the high DMS group had a worse outcome in the GSE22541 dataset (**Figure 1K**). The ROC curves for DMS at 1, 2, and 3 years in the GSE22541 dataset were 0.682, 0.659, and 0.661, respectively (**Figure 1L**).

### Identification of immune characteristic of the disulfidptosis metabolism-related signature

**Figure 2:**
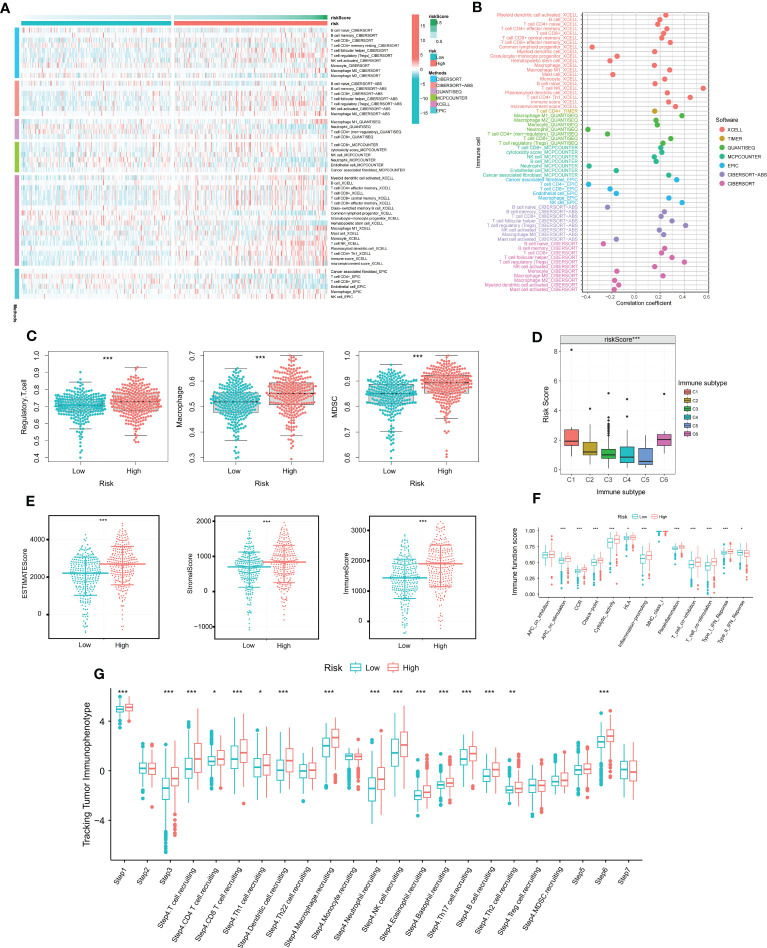
The immune characteristics of disulfidptosis related signature. **(A)** Distribution of immune infiltrating cells in Disulfidptosis related signature under various algorithms; **(B)** Correlation analysis of immune infiltrating cells and Disulfidptosis related signature under multiple algorithms; **(C)** Differential expression of immunosuppressive cells (Macrophage, Regulatory T cell, and MDSC) between high and low risk groups; **(D)** Differential expression of Disulfidptosis related signature in various immune subtypes; **(E)** Differential expression of tumor microenvironment scores (StromalScore, ImmuneScore, and ESTIMATEScore) between high and low risk groups; **(F)** Differential expression of immune functions scores between high and low Disulfidptosis related signature groups; **(G)** Differential expression of Disulfidptosis related signature in different Tracking Tumor immunophenotypes. *P-value <0.05; **P-value <0.01; ***P-value <0.001.

As ccRCC is known to be an immunoresponsive tumor with high heterogeneity and metastatic potential (14), we further investigated the prognostic model of the immune microenvironment characteristics. Multiple algorithms were utilized to evaluate the immune infiltration scores, and the distribution of immune infiltration cells between the two groups was visualized in a heat map (**Figure 2A**). The association between the immune infiltration cells and DMS was illustrated in **Figure 2B**, revealing a strong relationship between DMS and macrophages and Tregs. Furthermore, the expression levels of immunosuppressive cells, including Myeloid-derived suppressor cells (MDSCs), Regulatory T cells, and macrophages, were found to be significantly higher in the high DMS group (**Figure 2C**).

To assess the reliability of DMS in immunotyping, we examined the association between DMS and pan-cancer immune subtypes. The expression of DMS was higher in C1 and C6, but lower in C3, C4, and C5 (**Figure 2D**). Previous studies have reported that C3 may indicate a better prognosis, while C6 may be associated with a worse outcome. Additionally, we analyzed the potential relationship between DMS and tumor microenvironment scores. The ESTIMATE Score, Stromal Score, and Immune Score were significantly higher in the high DMS group (**Figure 2E**). Moreover, the scores of immune-related molecules such as Checkpoint, CCR, and Inflammation-promoting molecules were significantly elevated in the high DMS group compared to the low DMS group (**Figure 2F**).

To gain deeper insights into the role of immunocytes in ccRCC progression, we obtained immune activity scores at each corresponding step in ccRCC samples from the TIP database. The abundance of immune cells involved in the antitumor response exhibited significant differences between the two groups, as depicted in **Figure 2G**.

### Identification of clinicopathological characteristics of the disulfidptosis metabolism-related signature

**Figure 3:**
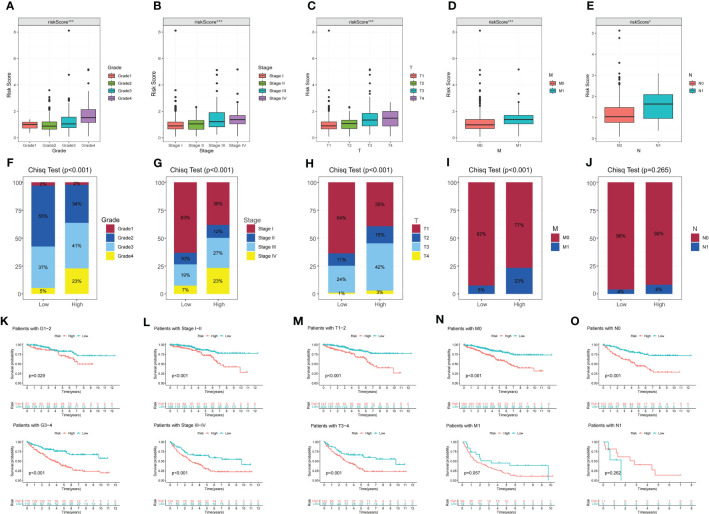
The correlation between disulfidptosis-related signature with clinicopathological features. **(A–E)** Different expressions of disulfidptosis-related signature among different clinicopathological subgroups. **(F–J)** Difference in the proportion of cases with different grades, stages, T, N and M stage between high and low-risk groups. **(K–O)** Validation of the prognostic efficacy of our model under the stratifications of different clinical parameters (histological Grade 1/2 and Grade 3/4, stage I/II and Stage III/IV, T1/2 and T3/4, N0 and N1, M0, and M1). *P-value <0.05; ***P-value <0.001.

To assess the prognostic features of DMS, we conducted an analysis of the association between DMS and clinicopathologic variables. Firstly, we examined the expression levels of DMS in different clinicopathologic variables. Our findings revealed significant differences in DMS expression among different clinicopathologic variables, including histological grade, pathological stage, and TMN stage. DMS was notably overexpressed in advanced clinicopathologic variables, indicating its potential as a prognostic indicator (**Figures 3A–E**). Additionally, we investigated the distribution of diverse clinicopathologic variables between the DMS groups. The results demonstrated significant differences in the proportions of advanced clinicopathologic variables, with a higher prevalence of advanced variables in the high DMS group (**Figures 3F–J**).

Furthermore, KM survival curves were utilized to assess the prognostic implications of DMS in different clinicopathologic variables. The results indicated that patients in the high DMS group had a worse prognosis across various clinicopathologic variables (**Figures 3K–O**). These findings further confirmed the significant negative correlation between DMS expression and the prognosis of ccRCC patients.

### Mutation and immunotherapeutic responses of the disulfidptosis metabolism-related signature

**Figure 4:**
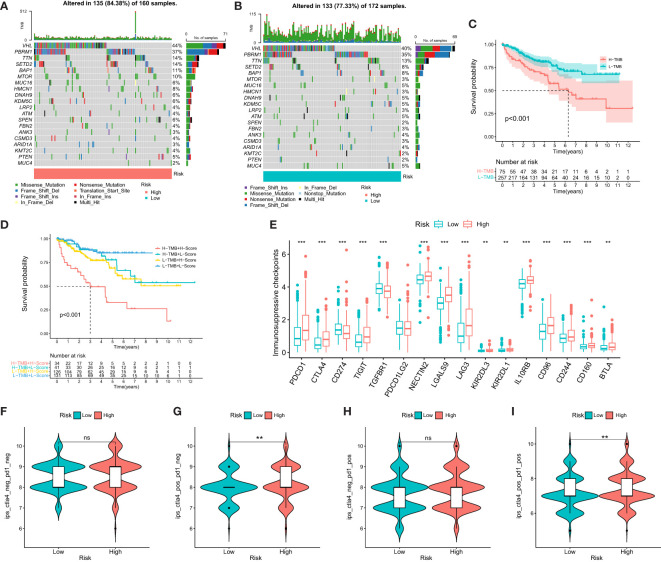
Mutational and immunotherapeutic characteristic of disulfidptosis-related signature. **(A, B)** Waterfall plots of somatic mutations in tumors in high and low risk groups; **(C)** KM survival analysis between high and low TMB; **(D)** Survival analysis of distinct groups stratified by both TMB and Disulfidptosis related signature; **(E)** Differences in the expression of immunosuppressive checkpoints between high and low risk groups; **(F–I)** Differential expression of anti-CTLA4 and/or anti-PD1 combination immunotherapy between high and low risk groups. **P-value <0.01; ***P-value <0.001.

To assess the relationship between TMB and DMS, we examined TMB changes in separate DMS groups. The high DMS group exhibited a mutation rate of 84.38% (135/160), while the low DMS group had a mutation rate of 77.33% (133/172). Interestingly, the top 20 genes with the highest mutation rates were consistent between the two DMS groups (**Figures 4A, B**). Furthermore, we observed a significant correlation between high TMB and poor prognosis (**Figure 4C**).

We then evaluated the combined prognostic value of DMS and TMB in ccRCC patients. The KM survival curve demonstrated that patients with both high TMB and high DMS (H-TMB+H-DMS) had the worst prognosis, while those with both low TMB and low DMS (L-TMB+L-DMS) had the best prognosis (**Figure 4D**). In addition, we analyzed the expression of immunosuppressive checkpoints between the two DMS groups. The results revealed that most of the immunosuppressive checkpoints were significantly overexpressed in the high DMS group (**Figure 4E**).

To further investigate the significance of DMS in evaluating the response to immunotherapy, we obtained immunotherapy data from the TCIA database. The probability of response to CTLA4-positive/PD-1-positive or negative treatment was higher in the high DMS group (**Figures 4F–I**). These findings suggest that patients with high-risk DMS may have a higher likelihood of responding to immunotherapy with CTLA4 and CTLA4+PD-1, leading to a better prognosis.

### Correlation between disulfidptosis metabolism-related signature and drug sensitivity

To explore the association between DMS and chemotherapeutic drug resistance in ccRCC, we performed IC50 analysis of nine major chemotherapeutic agents using the pRRophetic package. The results showed that the IC50 values of Bosutinib, Gefitinib, Nilotinib, Pazopanib, Rapamycin, Sunitinib, Vorinostat, Tipifarnib, and Temsirolimus were higher in the Low DMS group. This suggested that patients with higher DMS may have reduced sensitivity to these 9 drugs and that they may be more suitable for patients with lower DMS (**Figure S1**). These findings provided valuable insights for the selection of appropriate chemotherapeutic agents based on the DMS status of ccRCC patients.

### Prognosis and biological pathway characteristics of the disulfidptosis metabolism-related clusters

**Figure 5:**
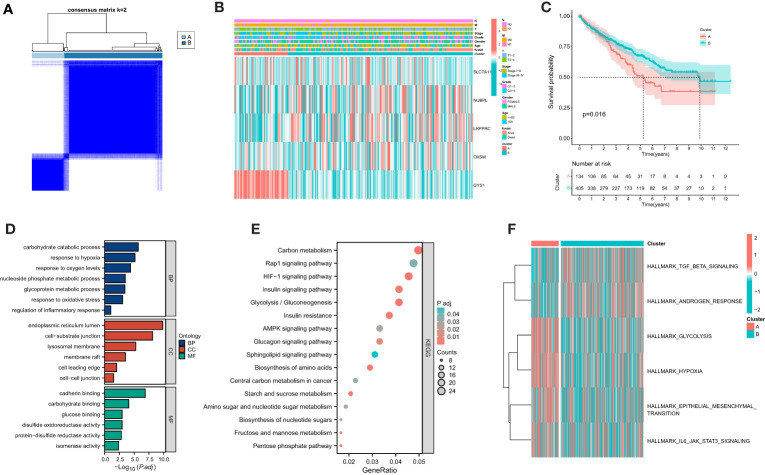
Establishment of disulfidptosis-related clusters and biological analysis. **(A)** Different disulfidptosis-related clusters of TCGA cohort were identified for k = 2. **(B)** Heatmap of the distribution of gene expression and clinicopathological variables. **(C)** Overall survival difference between cluster. **(D)** GO functional annotation analysis of differentially expressed genes (DEGs) between clusters. **(E)** KEGG pathway enrichment of DEGs between clusters. **(F)** GSVA enrichment analysis showed the enrichment distribution of biological pathways in clusters.

Based on the gene expression of 4 selected candidates, we utilized the ConsensuClusterPlus package to establish 2 disparate disulfidptosis metabolism-related clusters (**Figure 5A**). Heatmap showed the distribution of gene expression profiles and clinicopathological variables between different clusters (**Figure 5B**). The KM curve showed that the prognosis of cluster A was associated with poorer clinical outcomes (**Figure 5C**). As a further validation, the results of GO suggested that DEGs were mainly localized to inflammatory response, oxidative stress and hypoxia (**Figure 5D**). Simultaneously, KEGG analysis suggested that DEGs were focused on multiple metabolism-related pathways, HIF−1 signaling pathway and AMPK signaling pathway (**Figure 5E**). GSVA analysis revealed that cluster A was markedly localized to IL6 JAK STAT3 signaling and Hypoxia (**Figure  5F**).

### Identification of prognosis and clinicopathological characteristics of the SLC7A11

**Figure 6:**
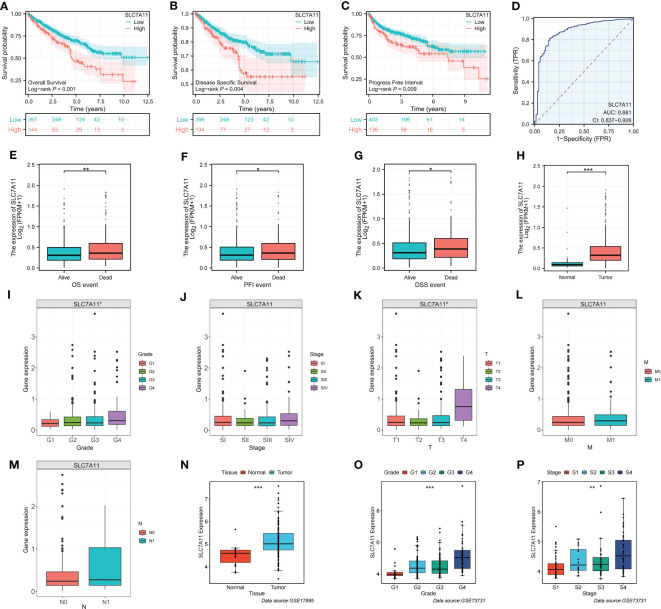
Identification of the clinicopathological and prognostic characteristics of SLC7A11. **(A–C)** Survival analysis of SLC7A11 in OS, PFI and DSS. **(D)** Time-dependent ROC curve of SLC7A11 in ccRCC. **(E–M)** Expression difference of SLC7A11 in different clinicopathological phases (**E**: OS, **F**: PFI, **G**: DSS, **H**: Tissue, **I**: Grade, **J**: Stage, **K**: T, **L**: M, **M**: N). **(N–P)** Validation of clinical characteristics of SLC7A11 in different GEO datasets (**N**: GSE17895; **O** and **P**: GSE73731). *P-value <0.05; **P-value <0.01; ***P-value <0.001.

SLC7A11 plays a crucial role in mediating disulfidptosis, a unique form of cell death characterized by the accumulation of intracellular disulfide molecules in cancer cells with dysregulated expression of the cystine transporter (7). In the context of ccRCC, we investigated the potential mechanism of action of SLC7A11 and its correlation with patient outcomes. Our analysis revealed that high expression of SLC7A11 was associated with worse OS, progression-free interval (PFI), and disease-specific survival (DSS) in ccRCC patients (**Figures 6A–C**). Additionally, SLC7A11 exhibited promising prognostic value, as demonstrated by the ROC curve analysis with an AUC of 0.881 (**Figure 6D**). Furthermore, SLC7A11 was significantly overexpressed in patients who experienced death and in tumor tissues (**Figures 6E–H**). We also explored the expression profile of SLC7A11 across different clinicopathological stages, revealing a positive correlation with histological grade, pathological stage, and TMN stage (**Figures 6I–M**). To validate our findings, we examined the expression profile of SLC7A11 in several clinical variables using GEO datasets, which confirmed its higher expression in advanced clinicopathological stages and cancer tissues (**Figures 6N–P**). These results highlighted the potential role of SLC7A11 as a prognostic marker and its association with disease progression in ccRCC.

### Identification of immunological characteristics of the SLC7A11

**Figure 7:**
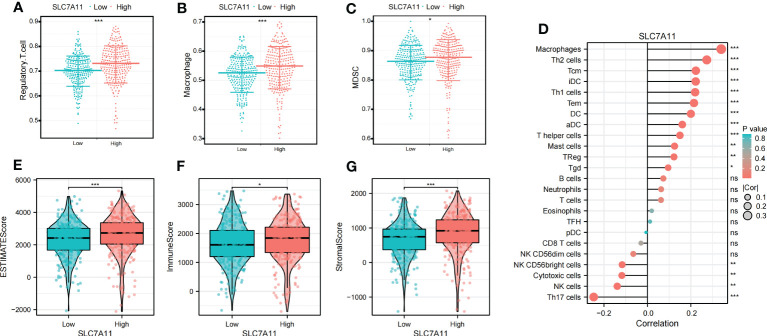
Identification of the immunoinfiltration characteristics of SLC7A11. **(A–C)** Differential expression of immunosuppressive cells (Macrophage, Regulatory.T.cell, and MDSC) between high and low SLC7A11 groups. **(D)** Correlation analysis between SLC7A11 and immune infiltrating cells; **(E–G)** Differential expression of tumor microenvironment scores (StromalScore, ImmuneScore, and ESTIMATEScore) between high and low SLC7A11 groups. *P-value <0.05; **P-value <0.01; ***P-value <0.001, ns, no significant.

To explore the relationship between SLC7A11 expression patterns and the immune microenvironment, we categorized ccRCC patients into high or low SLC7A11 expression groups based on the median value of SLC7A11 expression. Our analysis revealed that immunosuppressive cells, including Macrophage, MDSC, and Regulatory T cells, were significantly upregulated in the high SLC7A11 group (**Figures 7A–C**). Furthermore, the expression of SLC7A11 showed a positive correlation with the abundance of suppressive immunocytes, including Macrophage, MDSC, and Regulatory T cell (**Figure 7D**). We then assessed the immune microenvironmental characteristics associated with SLC7A11 and observed a positive correlation between SLC7A11 expression and immuneScore, stromalScore, and estimatScore (**Figures 7E–G**). These findings suggested that elevated SLC7A11 expression was associated with an immunosuppressive microenvironment in ccRCC, characterized by increased levels of immunosuppressive cells and altered immune and stromal scores.

### SLC7A11 promotes ccRCC cell proliferation *in vitro*

**Figure 8:**
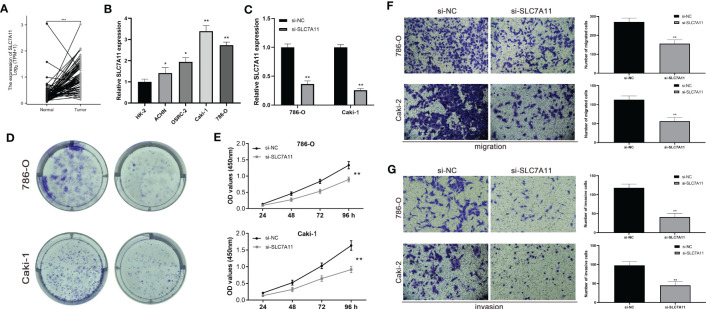
The expression levels of SLC7A11 promote ccRCC cell proliferation *in vitro*. **(A)** Expression of SLC7A11 in paired ccRCC tissues. **(B)** SLC7A11 mRNA expression in normal and ccRCC cell lines. **(C)** mRNA expression in SLC7A11 knockdown ccRCC cell lines. **(D, E)** Colony formation assay in si-SLC7A11 and control cells. **(F, G)** Transwell assays with SLC7A11 downregulated had fewer migrated cells. **(C, D)** Transwell assays with SLC7A11 downregulated had fewer invasive cells. *P-value <0.05; **P-value <0.01; ***P-value <0.001.

To validate the biological function of SLC7A11 in ccRCC, we first confirmed high expression of SLC7A11 in tumor tissues using paired samples from the TCGA database (**Figure 8A**). We then assessed the expression profile of SLC7A11 in ccRCC cell lines. **Figure 8B** showed that SLC7A11 was highly expressed in ACHN, OSRC-2, Caki-1, and 786-O cell lines compared to normal renal epithelial cells, with 786-O and Caki-1 exhibiting the highest expression levels. Therefore, we selected 786-O and Caki-1 cell lines for further study. The si-SLC7A11 used in the experiment effectively suppressed the expression of SLC7A11 (**Figure 8C**). Both CCK-8 and colony formation assays confirmed that knockdown of SLC7A11 significantly impaired the proliferation ability of 786-O and Caki-1 cells (**Figures 8D, E**). Furthermore, the transwell assay demonstrated that si-SLC7A11-treated 786-O and Caki-1 cells exhibited reduced migration and invasion capacities compared to the control group (**Figures 8F, G**). These results indicated that SLC7A11 played a role in promoting proliferation, migration, and invasion in ccRCC cells.

## Discussion

Considered the most common subtype of renal tumors, ccRCC exhibits high aggressiveness and poor prognosis (15). However, due to the asymptomatic nature of early stage ccRCC, there is a significant challenge in identifying effective predictors for early diagnosis and prognosis (15). As the understanding of cell death mechanisms expands, exploring and understanding these mechanisms has become crucial not only for carcinogenesis but also for tumor therapy (16). Triggering programmed cell death holds promise as a potential treatment strategy for tumors like ccRCC (17). Disulfidptosis, a novel cell death modality, differs from common types of cell death such as cuproptosis, ferroptosis, apoptosis, pyroptosis, and necrotic apoptosis. It is characterized by the abnormal accumulation of intracellular disulfides (7). Serum disulfide levels have been found to be significantly higher in breast cancer patients, suggesting a potential role in breast cancer pathogenesis (18). Disulfide, as an important modulator of oxidative metabolism, can influence crucial cellular activities, including tumor cell survival and metastasis (19). Therefore, disulfidptosis may represent a promising treatment strategy for ccRCC, as patients have the potential to respond to therapy and achieve better survival outcomes.

In our study, we aimed to predict treatment outcomes through risk stratification by establishing a prognostic signature consisting of four DMGs (LRPPRC, SLC7A11, OXSM, and GYS1) using multiple statistical analyses. The results, validated externally, demonstrated the excellent predictive performance of the signature. Building upon previous findings, we believed that uncovering the prognostic and therapeutic roles of genes involved in disulfidptosis will benefit the evaluation and treatment of ccRCC patients. Among the signature genes, LRPPRC has been implicated in promoting the tumorigenesis of bladder urothelial carcinoma by regulating intracellular ROS homeostasis and holds important prognostic significance (20). Enhancers at the OXSM locus have been identified as metastasis-specific enhancers in metastatic oral squamous cell carcinomas (OSCC), with OXSM playing a role in proliferation, invasion, and lipid synthesis in metastatic OSCC cells (21). GYS1 was found to be significantly overexpressed in ccRCC, leading to disturbances in glycogen metabolism and promoting ccRCC growth (22). SLC7A11 has been described as a key regulator of disulfidptosis, and a therapeutic strategy based on GLUT inhibition-induced disulfidptosis has shown promise in tumor patients with high SLC7A11 levels (23). Additionally, we noted that SLC7A11 was also a key regulator of ferroptosis, prompting us to conduct experiments to further explore its potentially oncogenic function. By knocking down SLC7A11 expression in ccRCC cell lines, we observed weakened proliferation, migration, and invasion, as demonstrated *in vitro*. The differential expression of these genes were tightly associated with tumor prognosis, partially explain the superiority of our signature.

In recent years, immunotherapy has emerged as a prominent treatment approach for ccRCC. Trials investigating immunotherapy targets in ccRCC have yielded significant insights, and disulfidptosis-related treatment may represent a promising strategy in the future. Given the critical role of the immune system in antitumor responses in ccRCC, we conducted a comprehensive assessment of immune infiltrate characteristics using various algorithms (24, 25). ccRCC is known for its unique immunogenicity, displaying a high degree of heterogeneity in CD8+ T cell infiltration, distinguishing it from many other solid tumor types (26, 27). Consistent with previous reports, we observed a strong correlation between CD8+ T cells and DMS, with higher levels of CD8+ T cell infiltration observed in the high-risk group. Additionally, Tregs, which possessed potent immunosuppressive properties, have been established as important contributors to ccRCC development (28). Our findings supported an inverse correlation between neutrophil expression and DMS. Neutrophils have gained significant attention in the fields of microbial infection and tumor development, with research indicating that neutrophils can exhibit an antitumor phenotype and directly or indirectly kill tumor cells, consistent with our results (29). In addition to immune infiltration analysis, the Immunophenoscore (IPS) algorithm has shown promising predictive performance for tumor immunotherapy efficacy (30). Our study suggested that high-risk patients may derive greater benefits from immunotherapy with CTLA4 and CTLA4+PD-1, indicating a more favorable response to immunotherapy. These findings further support the notion that patients with high-risk scores have a worse prognosis compared to those with lower scores.

To guide the development of clinical treatment strategies, we screened tyrosine kinase inhibitors, mTOR inhibitors, and histone deacetylase (HDAC) inhibitors from the pRRophetic package to assess their association with ccRCC resistance. Tyrosine kinase inhibitors are commonly used as first-line anti-angiogenic targeted therapies, inhibiting VEGF and its receptor (VEGFR) signaling in patients with metastatic RCC (31). mTOR, a highly conserved protein kinase, regulates RCC cell metabolism and proliferation through the PI3K and Akt pathways, making mTOR inhibitors an important therapeutic option (32). HDAC inhibitors induce degradation of hypoxia-inducible factor-1 and -2α, demonstrating antitumor effects (33, 34). Our analysis showed that the IC50 of the 9 chemotherapeutic drugs was lower in the high DMS group, indicating that patients in this group exhibited higher sensitivity to chemotherapy drugs.

The molecular heterogeneity of ccRCC reflects its complexity and has implications for its occurrence and development, as well as guiding therapeutic decisions. In our study, we successfully identified two molecular subtypes of ccRCC based on signature genes, which may have specific clinical implications. GO analysis revealed that the DEGs were primarily associated with oxidative stress and hypoxia. KEGG analysis indicated their involvement in HIF-1 signaling pathway and AMPK signaling pathway. Oxidative stress-induced structural and functional changes in proteins, lipids, and nucleic acids have been observed in ccRCC (35). Furthermore, ccRCC is known to be associated with a strong state of oxidative stress, which is directly contributes to its occurrence and development (36). Hypoxia-mediated pathways have also been implicated in tumorigenesis, particularly in ccRCC (37). AMPK, a cellular energy sensor, regulates metabolism, cell growth, and apoptosis, and its dysregulation is strongly associated with tumors, including ccRCC (38). Our study highlighted the potential role of these molecular pathways in ccRCC heterogeneity, suggesting a correlation between disulfidptosis and these pathways, which may promote the development and progression of ccRCC and serve as potential therapeutic targets.

While there have been numerous studies exploring the relationship between gene-regulated cell death and ccRCC, the role of disulfidptosis, as a novel cell death modality, has not been investigated extensively. In this study, we shed light on the prognostic and immunological role of genes involved in disulfidptosis in ccRCC, providing valuable insights for prognosis and therapeutic guidance. However, it is important to acknowledge certain limitations. Firstly, our study primarily focused on Western populations, which may introduce some regional differences. Secondly, further experimental research is necessary to validate our findings and elucidate the underlying mechanisms.

## Data availability statement

The original contributions presented in the study are included in the article/**Supplementary Material**. Further inquiries can be directed to the corresponding author.

## Ethics statement

All the patients provided written informed consent, and the protocol was approved by ethical committee of The First Affiliated Hospital of Nanjing Medical University.

## Author contributions

DZ, XiZ, ZL, and TH designed the study and performed all analyses. XX, KZ, XuZ, and XR wrote the manuscript. CQ revised the manuscript. All authors contributed to the article and approved the submitted version.

## Supplementary material

Supplementary Figure 1 Drug sensitivity of disulfidptosis-related signature. Sensitivity analysis for Bosutinib **(A)**, Gefitinib **(B)**, Nilotinib **(C)**, Pazopanib **(D)**, Rapamycin **(E)**, Sunitinib **(F)**, Vorinostat **(G)**, Tipifarnib **(H)** and Temsirolimus **(I)** between low and high disulfidptosis-related signature.
