# Supplementary figures and images for: An integrative multi-omics analysis based on disulfidptosis-related prognostic signature and distinct subtypes of clear cell renal cell carcinoma
Source: Front Oncol. 2023 Jun 23;13:1207068. doi: 10.3389/fonc.2023.1207068 (PMC10327293; doi:10.3389/fonc.2023.1207068)

A

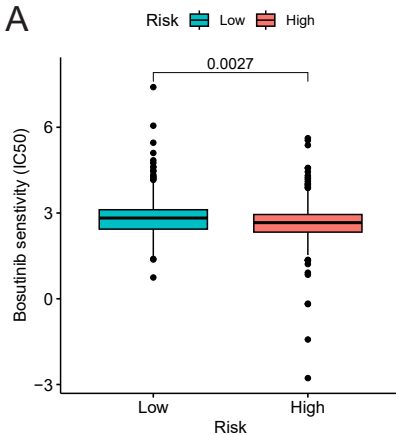

B

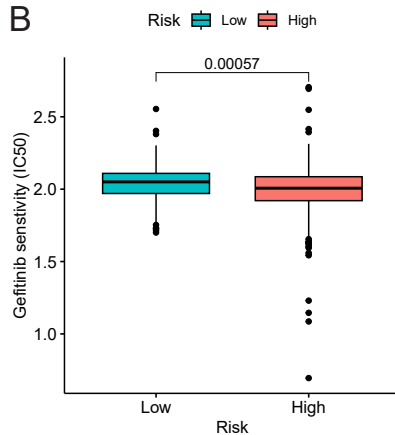

C

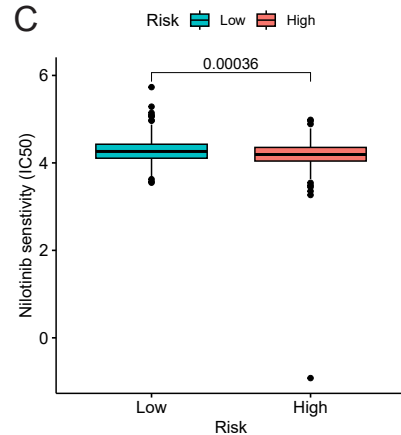

D

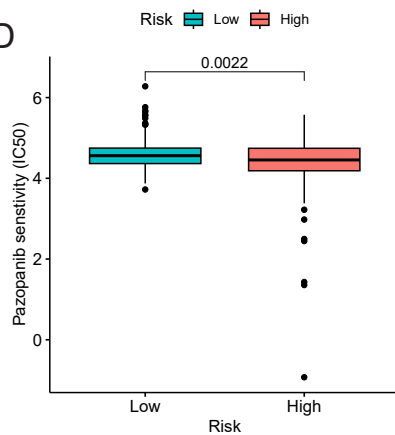

E

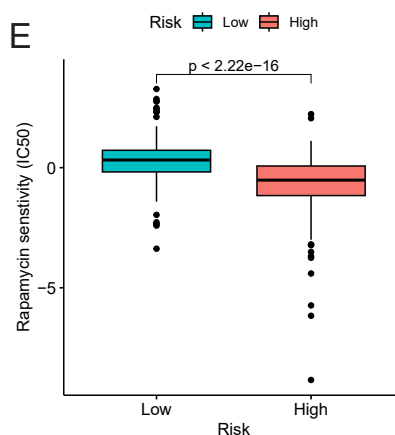

F

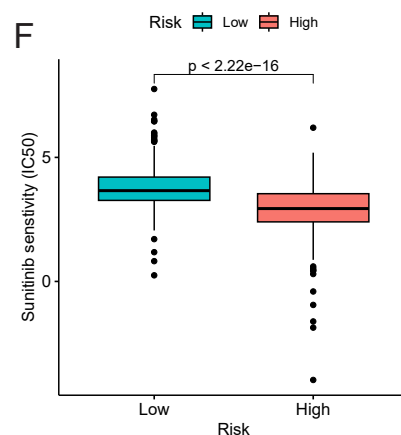

G

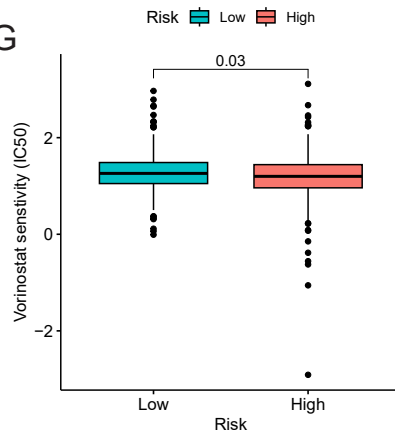

H

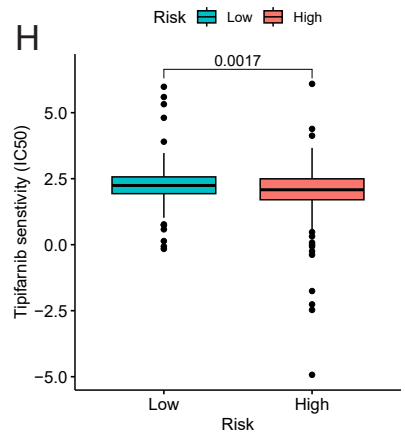

I

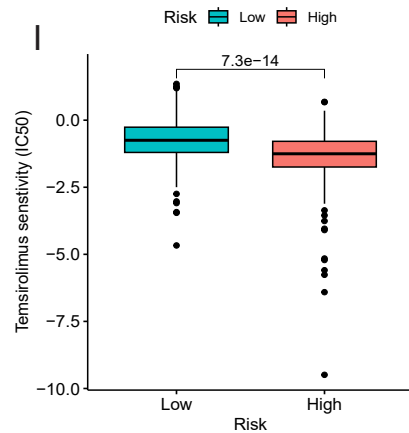

Supplement: Supplementary Figure 1 — Drug sensitivity of disulfidptosis-related signature. Sensitivity analysis for Bosutinib (A), Gefitinib (B), Nilotinib (C), Pazopanib (D), Rapamycin (E), Sunitinib (F), Vorinostat (G), Tipifarnib (H) and Temsirolimus (I) between low and high disulfidptosis-related signature. [file Image_1.pdf]
